# Refractive predictive errors using Barrett II, Hoffer-Q, and SRKT formulae for pediatric IOL implantation

**DOI:** 10.1007/s00417-024-06401-4

**Published:** 2024-02-15

**Authors:** Or Shmueli, Nur Azem, Ana Navarrete, Milka Matanis-Suidan, Ran David, Hadas Mechoulam, Irene Anteby

**Affiliations:** Department of Ophthalmology, Hadassah Medical Center, The Hebrew University of Jerusalem, 9112001 Ein-Karem, Jerusalem Israel

**Keywords:** Intra-ocular lens power calculation, Barrett Universal II formula, Pediatric cataract, Children, Accuracy, Predictive error

## Abstract

**Purpose:**

To compare the accuracy of the Barrett II universal (BU II) formula, Hoffer-Q, and SRKT formulae following lensectomy and IOL implantation in a large pediatric cohort.

**Methods:**

Retrospective study of children who underwent lensectomy and IOL implantation between 2015 and 2023 at Hadassah-Hebrew University Medical Center, Jerusalem, Israel.

**Results:**

One hundred and fifty-one eyes of 104 children aged 6.0 ± 3.9 years were included. The mean prediction error (PE) was − 0.08 ± 1.54 diopters (D) with BU II, 0.24 ± 1.46 D with Hoffer-Q, and 0.71 ± 1.92 D with SRKT (*P* = 0.10). In eyes with axial length (AL) < 22 mm, BU II and Hoffer-Q had a smaller PE than SRKT (*P* = 0.024). In eyes with AL ≥ 22 mm, BU II had a smaller PE than Hoffer-Q (*P* = 0.048).

In children 24 months or older at surgery, BU II had a smaller PE than SRKT and Hoffer-Q (*P* = 0.012). However, in younger children, no difference was found between the formulae (*P* = 0.61).

For mean *k*-values ≥ 44.5 D, BU II and Hoffer-Q had a smaller PE than SRKT (*P* = 0.002). An absolute prediction error < 1.0 D was obtained with BU II in 66% of eyes and SRKT in 35% (*P* = 0.01).

**Conclusions:**

The BU II formula performed well with a small prediction error. No significant difference in PE was detected overall between the formulae. However, only BU II demonstrated a stable prediction error at varying axial lengths, K-readings, and ages. As the biometric parameters of the developing eye change with growth, the BU II formula offers a reliable and stable option for pediatric IOL calculation.

**Supplementary Information:**

The online version contains supplementary material available at 10.1007/s00417-024-06401-4.


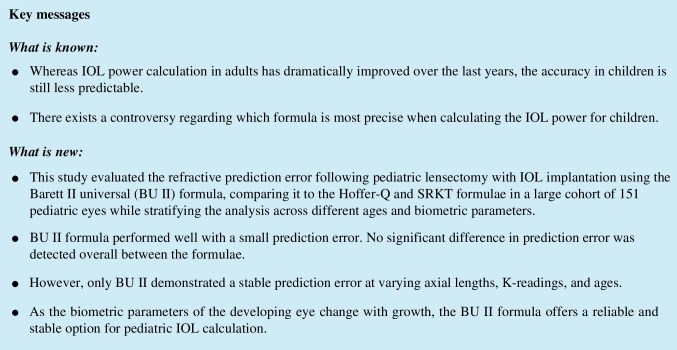


## Introduction

Congenital and childhood cataracts constitute a significant cause of visual disability in children and are responsible for 5–20% of pediatric blindness globally [1]. When a cataract significantly affects vision or visual development, it is surgically removed with or without intra-ocular lens (IOL) implantation. There exists a controversy regarding which is the most suitable target IOL power and which formula is most precise when calculating the IOL power for children. Whereas the perfection of IOL power calculation in adults has dramatically improved over the last years, the accuracy in pediatric eyes is generally poorer and more unpredictable.

Barrett II (BU II) formula shows superior accuracy over early-generation formulas when calculating the IOL power in adult eyes [2, 3]. In a study by Cooke et al. [2], using the Barrett II universal (BU II) formula resulted in a prediction error (PE) smaller than ± 1.0 diopter in 99% of adult eyes.

The challenges when calculating the IOL power in a pediatric population are numerous. The unique features to consider in these eyes include smaller axial length (AL), keratometry, and anterior chamber depth. In addition, the effective lens position may be affected by the need for anterior vitrectomy in young children [4, 5]. Currently, there are no IOL formulas adapted to children. Pediatric cataract surgeons commonly use the Sanders-Retzlaff-Kraff Theoretical formula (SRKT), Hoffer-Q, and Holladay formulae. The accuracy of these formulas is worse when compared to adult IOL calculations, with 25–26% of pediatric eyes having PE greater than two diopters at 2–6 months postoperatively [6].

Several studies address using the BU II formula when calculating the IOL power in children (see Table 1). Most show BU II to be comparable or superior to most other tested formulae, especially compared to older generations such as Hoffer-Q and SRKT. Some studies found that BU II had greater stability and reliability of accuracy throughout changing biometric parameters [7]. In contrast, others found BU II less reliable than other formulae (e.g., SRKT, Emmetropia Verifying Optical formula [EVO]) for shorter eyes [8–10].

**Table 1:** Review of studies comparing Barrett II Universal with other formulae for pediatric IOL calculation

Study authors, year	Number of eyes; mean or median (SD^1^ or range) age	Mean or median APE^2^ (SD or confidence interval; D)	% of eyes with PE^3^ < ± 1.0 D^4^	Main findings and additional comments
Yilmaz et al. 2022 [9]	70 eyes 3–15 years	BU II^5^: 0.64 ± 0.73 Hoffer-Q: 0.65 ± 0.64 SRK/T^6^: 0.67 ± 0.65 HOLLADAY 1: 0.67 ± 0.73 Haigis: 1.06 ± 0.84	BU II: 82.9% Hoffer-Q: 81.4% SRK/T: 78.6% HOLLADAY 1: 78.6% Haigis: 57.1%	BU II formula had the lowest APE in eyes with average AL^7^ (0.64 ± 0.73) and in eyes with AL < 22 mm (0.47 ± 0.54) SRK/T and Holladay 1 formulas had the lowest APE in eyes with AL < 22 mm (0.77 ± 0.68 and 0.79 ± 0.71, respectively)
Rastogi et al. 2022 [11]	60 eyes Mean 8.53 (5–16) years	BU II: 0.64 ± 0.73 Hoffer-Q: 0.71 ± 0.65 SRK/T: 0.8 ± 0.75 HOLLADAY 1: 0.7 ± 0.72	BU II: 80% Hoffer-Q: 83.33% SRK/T: 73.33% HOLLADAY 1: 80%	BU II had the lowest MAE, although this was not statistically significant (*p* = 0.176)
Rastogi et al. 2022 [12]	99 eyes Mean 6.5 (4–18) years	BU II: 1.24 ± 1.20 Hill-RBF: 1.08 ± 1.00 Hoffer-Q: 1.25 ± 1.06 SRK/T: 1.25 ± 1.10 HOLLADAY 1: 1.28 ± 1.01	BU II: 66.7% Hill-RBF: 73.7% Hoffer-Q: 46.6% SRK/T: 60% HOLLADAY 1: 50%	Hill-RBF, BU II, and SRKT formulae did not show statistically significant MAE
Rajaan-Nakhli 2019 [13]	44 eyes Median 2.85 (2.04–6.14) years	BU II: 1.49 ± 1.08 Olsen: 1.58 ± 1.16 Hoffer-Q: 1.48 ± 1.11 SRK/T: 1.43 ± 1.09 HOLLADAY 1: 1.43 ± 0.99 Haigis: 2.23 ± 1.39 Holladay II: 1.52 ± 1.08	BU II: 38.6% Olsen: 31.8% Hoffer-Q: 36.4% SRK/T: 38.6% HOLLADAY 1: 36.4% Haigis: 15.9% Holladay II: 31.8%	Four new-generation formulae (Haigis, Holladay II, Olsen, and Barrett Universal II) were comparable to four standard formulas (Holladay I, Hoffer Q, SRK/T, and SRKII)
Chang et al. 2020 [8]	68 eyes Mean 2.86 (± 2.0) years	BU II: 0.95 ± 0.78 Olsen: 1.19 ± 0.98 Hoffer-Q: 1.01 ± 0.72 SRK/T: 0.93 ± 0.77 HOLLADAY 1: 1.00 ± 0.73 Haigis: 1.11 ± 1.09 Holladay II: 1.23 ± 0.92	BU II: 66.2% Olsen: 54.4% Hoffer-Q: 61.8% SRK/T: 66.2% HOLLADAY 1: 57.4% Haigis: 58.8% Holladay II: 51.5%	In children younger than two years old or with AL ≤ 21 mm, SRK/T formulas were relatively accurate (with 55% of eyes having APE < 1 D, while BU II had 45–50% of eyes with APE < 1 D), while BU II was better in patients older than two years or with AL > 21 mm (with 80% of eyes having APE < 1 D)
Eppley et al. 2021 [7]	64 eyes Median 5.4 (3–16) years	BU II: 0.79 (0.51, 0.88) Hoffer-Q: 0.88 (0.68, 1.04) SRK/T: 0.86 (0.55, 1.02) HOLLADAY 2: 0.81 (0.63, 1.06)	BU II: 67% Hoffer-Q: 59% SRK/T: 61% HOLLADAY 2: 58%	BU II accuracy was comparable to Holladay 2 and SRKT formulae, although BU II showed greater stability and reliability of accuracy throughout changing biometric parameters
Elbaz et al. 2022 [14]	62 eyes Median 6.2 (0.9–17.5) years	BU II: 0.89 ± 1.0 Hoffer-Q: 0.87 ± 1.03 SRK/T: 0.95 ± 0.98 HOLLADAY 1: 0.92 ± 1.02 Haigis: 0.96 ± 1.10	BU II: 69.7% Hoffer-Q: 69.7% SRK/T: 68.2% HOLLADAY 1: 71.2% Haigis: 66.7%	BU II had comparable accuracy to other tested formulae and outperformed the SRK/T formula when calculating IOL power within the 0.5 D range from target refraction in pediatric eyes (51.5% vs 31.8%; *P* = 0.002)
Reitblat et al. 2022 [15]	62 eyes Median 6.2 (0.9–17.5) year	BU II: 0.88 ± 1.0 Kane: 0.91 ± 1.04 Hoffer-Q: 0.88 ± 1.05 SRK/T: 0.97 ± 1.0 HOLLADAY 1: 0.94 ± 1.05 Haigis: 0.98 ± 1.13	BU II: 74.1% Kane:70.4% Hoffer-Q: 74.1% SRK/T: 70.4% HOLLADAY 1: 70.4% Haigis: 70.4%	BU II had comparable accuracy to other tested formulae, including Kane, Haigis, Hoffer Q, Holladay 1, and SRKT
Lin et al. 2022 [10]	110 eyes Mean 3.1 (± 1.9) years	BU II: 0.87 ± 0.77 SRK/T: 0.95 ± 0.73 EVO^8^: 0.85 ± 0.65 Kane: 0.88 ± 0.88 Haigis: 1.16 ± 0.98	BU II: 68.2% SRK/T: 61.8% EVO: 68.2% Kane: 64.6% Haigis: 55.5%	BU II, EVO, and Kane formulas were relatively accurate in children older than 24 months and with AL > 21 mm, while in children younger than 24 months and with AL ≤ 21 mm, EVO was mildly more accurate

A review of studies comparing Barrett II Universal with other formulae for pediatric IOL calculation. The compared criteria include absolute refractive prediction error (APE) and the percentage of eyes with refractive prediction error (PE) within ± 1.0 diopter. Continuous data are presented as the mean ± standard deviation (minimum–maximum) or median (range or confidence interval). Categorical data are presented as proportions

*SD*^1^, standard deviation; *APE*^2^, absolute refractive prediction error; *PE*^3^, refractive prediction error; *D*^4^, diopters; *BU II*^5^, Barrett Universal II formula; *SRKT*^6^, Sanders-Retzlaff-Kraff Theoretical formula; *AL*^7^, axial eye length; *EVO*^8^, Emmetropia Verifying Optical formula

*P-value < 0.05 was considered significant

Here, we aimed to evaluate the refractive prediction error one month following pediatric lensectomy with IOL implantation using the BU II formula, comparing it to the Hoffer-Q and SRKT formulae in a large cohort of children while stratifying the analysis across different ages and biometric parameters.

## Methods

### Study design, patient selection, and data collection

In this retrospective study, we collected the baseline and follow-up data of children who underwent lensectomy and IOL implantation during the years 2015 to 2023 at the Center for Pediatric Ophthalmology and Strabismus at the Hadassah-Hebrew University Medical Center in Jerusalem, Israel. This study was performed in accordance with the Declaration of Helsinki and was approved by the Institutional Review Board (IRB)/Ethics Committee (approval number HMO-371–19). Informed consent was not required due to the retrospective study design and anonymous data analysis.

We included children less than 18 years of age who underwent cataract extraction with IOL implantation and were followed for at least one month. We excluded children without sufficient documentation of biometry, predicted post-operative refraction, or actual post-op refraction. Additional exclusion criteria included associated uveitis, corneal transplant, or anterior segment dysgenesis.

Demographic data was extracted from the children’s electronic medical records, including gender, age, laterality, history of trauma or persistent fetal vasculature, primary or secondary IOL insertion, in the bag or sulcus IOL location, IOL type (Bausch and Lomb MX60; Sensar AR40E 3 piece; Medennium Matrix 3 piece; Alcon acrysof IQ; Alcon 3 piece; PMMA [Polymethylmetacrylate]), axial length, keratometry, the formula used for IOL calculation, method of axial length acquisition (IOL master 500 or contact A-scan), and pre and post-operative refraction one month after surgery.

### Measured parameters

In uncooperative or young children, biometry was obtained during anesthesia: biometry (axial length and keratometry) by a hand-held autorefractor keratometer (Retinomax K-plus 3, Nidek Co Ltd., Tokyo, Japan) and contact A-scan biometry (Biometer AL-100; Tomey, Germany). In older children, IOL master 700 (Carl Zeiss Meditec AG, Jena, Germany) was used to obtain biometry measurements.

Pre (up to 1 month before surgery) and post (1–3 months following surgery) refractive errors were determined by cycloplegic retinoscopy. The refractive error was converted to spherical equivalent (SE).

### Surgical protocol

All children underwent lensectomy through a limbal approach with an IOL placed in the bag or the sulcus. The surgeries were performed by one surgeon (I.A.) using the same surgical technique. Posterior capsulectomy and anterior vitrectomy were performed in all children under eight years old. The IOL implanted (Bausch and Lomb MX60; Sensar AR40E 3 piece; Medennium Matrix 3 piece; Alcon acrysof IQ; Alcon 3 piece; or PMMA [Polymethylmetacrylate) and the formulae used to determine the IOL power depended upon surgeon discretion (see Table 2 for further details).

**Table 2:** Baseline patient characteristics

Factor	Barrett (*N*^1^ = 100)	Hoffer-Q (*N* = 31)	SRKT (*N* = 20)	*P*-value
Age at IOL^2^ implantation (months)				
Mean	75.2 ± 48.3 (10–195)	65.4 ± 43.6 (10–132)	65.0 ± 43.2 (14–162)	0.46
Median	74.0 (32.25–102.75)	72.0 (27.0–101.0)	57.5 (27.3–91.0)	0.51
Gender (%male)	55%	51.6%	80%	0.089
Bilateral cataract (% of eyes)	72%	87%	80%	0.22
Congenital/developmental				
cataract (%)	92%	100%	90%	0.45
Traumatic Cataract (%)	6%	0%	10%	
Associated with PFV^3^ (%)	2%	0%	0%	
Axial length (mm)				
Mean	21.8 ± 1.2 (19.5–25.9)	20.8 ± 1.2 (18.4–22.7)	22.5 ± 2.3 (18.2–28.0)	< 0.0001*
Median	21.6 (20.8–22.7)	20.8 (19.7–22.0)	22.3 (21.4–23.5)	0.001*
Pairwise comparison	Difference of means: Barrett-SRKT (*P* = 0.11), Barrett-Hoffer-Q (***P*** **= 0.002**), Hoffer-Q-SRKT (***P*** **< 0.0001**) Difference between medians: Barrett-SRKT (***P*** **= 0.05**), Barrett = Hoffer-Q (*P* = 0.164), Hoffer-Q-SRKT (***P*** = **0.008**)			
Average K^5^ (D^4^)				
Mean	44.1 ± 2.0 (39.7–48.3)	44.6 ± 1.8 (39.0–47.8)	44.2 ± 2.05 (40.3–47.4)	0.85
Median	44.7 (43.4–45.7)	44.4 (43.6–45.4)	43.95 (42.93–46.25)	0.76
Secondary IOL insertions (%)	9%	12.9%	30%	0.038*
IOL location (% located in the bag)	82%	90.3%	65%	0.074
IOL type (%)	B&L MX60 (80%) AR40E 3 piece (10%) Matrix 3 piece (7%) Acrysof IQ (1%) Alcon 3 piece (1%) PMMA (1%)	Acrysof IQ (90.4%) Alcon 3 piece (9.6%)	Acrysof IQ (60%) Alcon 3 piece (40%)	**< 0.0001**

Continuous data are presented as the mean ± standard deviation (minimum–maximum) and median (interquartile range). Categorical data are presented as proportions

*N*^1^, number of eyes; *IOL*^2^, intra-ocular lens implant; *PFV*^3^, persistent fetal vasculature; *D*^4^, diopters; *Average K*^5^, average corneal refractive power

*P-value < 0.05 was considered significant

### Study group and outcomes

We divided the eyes into three groups according to the formula used for IOL power calculation: Barrett II universal, Hoffer-Q, or Sanders-Retzlaff-Kraff Theoretical (SRK/T). The choice of formula depended on the surgeon’s preference and the time of surgery. In general, Hoffer-Q was chosen for eyes < 22 mm and SRKT was chosen for larger eyes. From the year 2018, BU II was used in the calculation of all IOL implantations. The placement of IOL in the sulcus was only in eyes with secondary implantations. An adjustment of 0.5–1.0 diopters decrease in IOL power was made in this case.

The predicted post-operative refraction was based on the AAO guidelines, considering the changes in growing pediatric eyes [16].

The predicted post-operative refraction was extracted from the chart review and verified based on the biometry results. Manufacturer constants were used for IOL calculations for both ultrasound and optical biometry.

Prediction error (PE) was calculated using the following formula: [prediction error = predicted spherical equivalent– actual post-operative spherical equivalent]. Absolute prediction error (APE) was obtained by converting PE into an absolute value.

### Statistical analysis

We analyzed data using SPSS software version 26 (IBM). The normality distribution of variables was tested by the Shapiro–Wilk test. Descriptive statistics of continuous variables are presented as mean ± standard deviation (SD) and median ± interquartile range (IQR).

Although some works suggest that the comparison of median PE/APE is better than the comparison of means, most publications have focused on means, as did the present work [7–12, 14, 15, 17]. Comparison of means was performed with the Kruskal–Wallis or analysis of variance (ANOVA) tests dependent on the normality of the distribution of variables in the study groups. A comparison of medians was performed with the median test for k samples. Descriptive statistics of categorical variables are presented as percentages. Categorical data were compared with the Fischer exact test.

The percentage of eyes within an APE of 0.25 D, 0.50 D, or 1.00 D was also compared across the various IOL formulae. The association of different variables with an APE > 1 D was assessed first using univariate multinomial logistic regression analysis. Subsequently, variables that demonstrated a significant effect in univariate analysis were included in a multivariate analysis using stepwise linear multinomial logistic regression. The exact process was also performed for assessing associations of different variables with an APE > 0.75, APE > 1.25, and APE > 1.5 (these results are available as Supplementary information tables).

### Sub-group analyses

Sub-group analysis was done for APE of the different formulae stratified by axial length < 22 mm or ≥ 22 mm, age < 24 months or ≥ 24 months, and average keratometry < 44.5 diopters or ≥ 44.5 diopters. These cutoff values were selected by considering both meaningful developmental differences (below or above 24 months) and a sample size of at least *N* ≥ 4 for each sub-group to evaluate for statistically significant differences.

Additional sub-group analysis was done for APE of the different formulae stratified by surgical method (performance or anterior vitrectomy and posterior capsulotomy as part of lensectomy with primary IOL implantation) and method of axial length acquisition (IOL master 500 or contact A-scan).

## Results

### Baseline characteristics

151 eyes of 104 children who underwent lensectomy with IOL implantation were included. At surgery, 57.6% were males with a mean age of 6.0 ± 3.9 years (range: 10 months–16.3 years).

Table 2 compares the baseline characteristics between the 3 study groups.

The Hoffer-Q group had a shorter mean axial length, and the SRKT group included more secondary IOL implantations. The IOLs used in the Hoffer-Q and SRKT groups were Acrysof IQ and Alcon 3-piece, while the main IOLs used in the Barrett group were the B&L MX-60, AR40E 3-piece, and Matrix 3-piece.

### Prediction errors according to the IOL formula

**Fig. 1:**
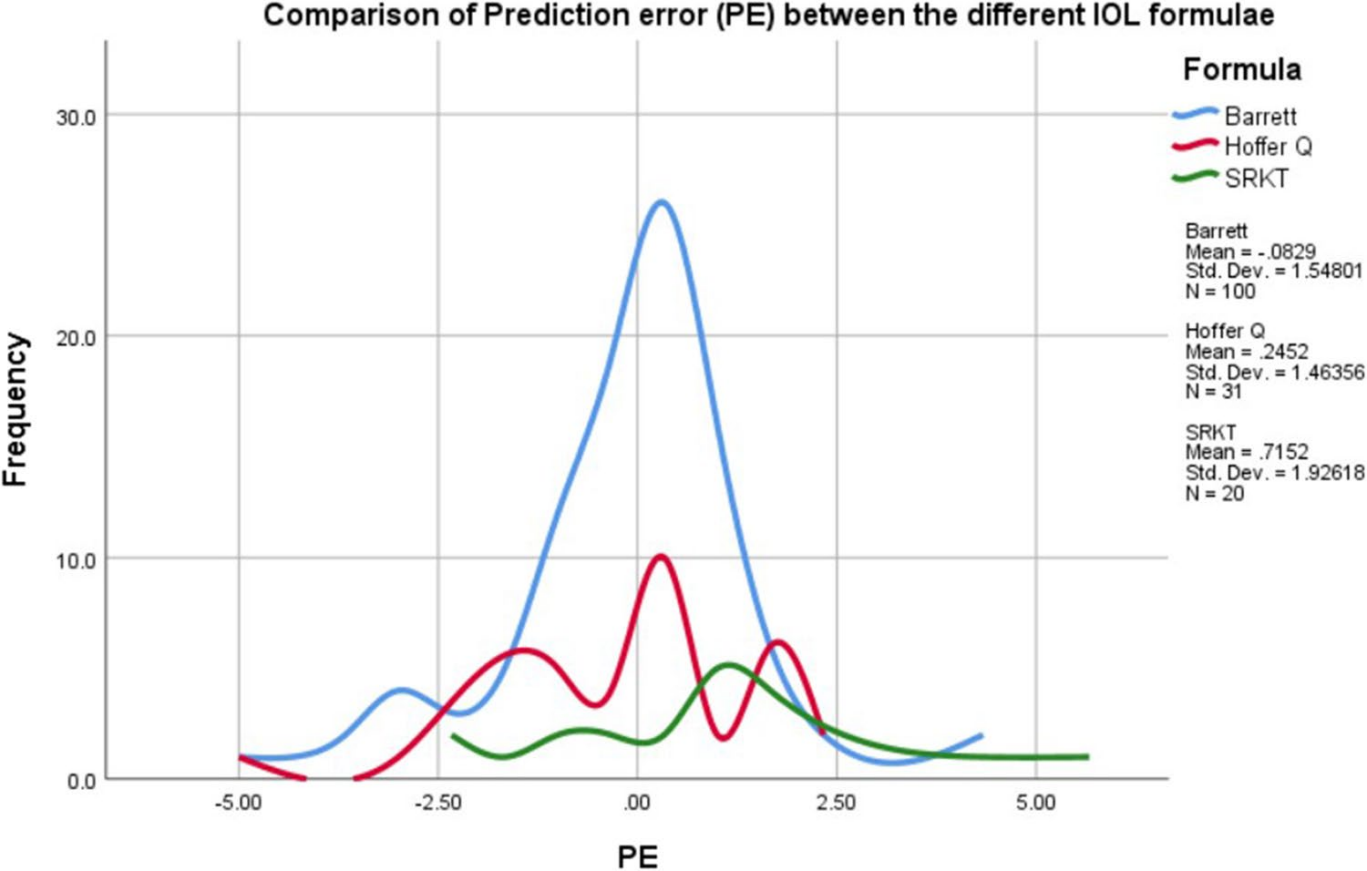
Comparison of prediction error (PE) between the different IOL formulae. The mean refractive prediction error was − 0.08 ± 1.54 with BU II, 0.24 ± 1.46 with Hoffer-Q, and 0.71 ± 1.92 with SRKT (*P* = 0.10). Data are presented as the mean ± standard deviation. N = number of eyes; PE = refractive prediction error; IOL = intra-ocular lens implant

The mean refractive prediction error was − 0.08 ± 1.54 diopters (D) using BU II, 0.24 ± 1.46 D using Hoffer-Q, and 0.71 ± 1.92 D using SRKT (*P* = 0.10). A comparison of the mean prediction error (PE) is presented in Fig. 1 and Table 3.

**Table 3:** Refractive prediction error comparing the different formulas

Factor	Barrett	Hoffer-Q	SRKT	*P*-value
Whole sample PE^2^	− 0.08 ± 1.54	− 0.08 ± 1.54	0.71 ± 1.92	0.10
	*N*^1^ = 100	*N*^1^ = 100	*N* = 20	
Axial length < 22 mm				
Mean	0.23 ± 1.56	0.09 ± 1.56	1.84 ± 2.04	0.024*
*N*	*N* = 59	*N* = 24	*N* = 8	
Pairwise comparison	Barrett-SRKT (***P*** **= 0.02**), Barrett-Hoffer-Q (*P* = 0.92), Hoffer-Q-SRKT (***P*** **= 0.02**)			
Axial length ≥ 22 mm				
Mean	− 0.54 ± 1.41	0.77 ± 0.94	− 0.03 ± 1.48	0.048*
*N*	*N* = 41	*N* = 7	*N* = 12	
Pairwise comparison	Barrett-SRKT (*P* = 0.20), Barrett-Hoffer-Q (***P*** **= 0.02**), Hoffer-Q-SRKT (*P* = 0.27)			
Age < 24 months				
Mean	0.54 ± 2.18	− 0.81 ± 2.33	0.82 ± 2.47	0.61
*N*	*N* = 15	*N* = 7	*N* = 4	
Age ≥ 24 months				
Mean	− 0.19 ± 1.39	0.55 ± 0.96	0.68 ± 1.86	0.012*
*N*	*N* = 85	*N* = 24	*N* = 16	
Pairwise comparison	Barrett-SRKT (***P*** **= 0.056**), Barrett-Hoffer-Q (***P*** **= 0.056**), Hoffer-Q-SRKT (*P* = 0.95)			
Average K^3^ < 44.5 diopters				
Mean	0.10 ± 1.45	0.13 ± 0.82	2.03 ± 1.91	0.002*
*N*	*N* = 52	*N* = 15	*N* = 8	
Pairwise comparison	Barrett-SRKT (***P*** = **0.002**), Barrett-Hoffer-Q (*P* = 0.99), Hoffer-Q-SRKT (***P*** = **0.008**)			

Continuous data are presented as the mean ± standard deviation

*N*^1^, number of eyes; *PE*^2^, refractive prediction error; *Average K*^3^, average corneal refractive power

*P-value < 0.05 was considered significant

In short eyes, defined as AL < 22 mm, BU II and Hoffer-Q had smaller PE than SRKT (0.23 ± 1.56 D, 0.09 ± 1.56 D, and 1.84 ± 2.04 D, respectively; *P* = 0.024). In longer eyes with AL ≥ 22 mm, BU II had a smaller PE when compared to Hoffer-Q (− 0.54 ± 1.41 D and 0.77 ± 0.94 D, respectively; *P* = 0.048). No significant difference in PE was seen between SRKT and BUII or SRKT and Hoffer-Q.

In children 24 months or older, eyes with IOL implanted using the BU II had a smaller PE than eyes using SRKT and Hoffer-Q (− 0.19 ± 1.39 D, 0.55 ± 0.96 D, and 0.68 ± 1.86 D, respectively; *P* = 0.012). There was no difference in PE in children younger than 24 months (0.54 ± 2.18 D, − 0.81 ± 2.33 D, and 0.82 ± 2.47 D, respectively; *P* = 0.61).

At mean *k*-values ≥ 44.5 diopters, BU II and Hoffer-Q had a PE lower than SRKT (0.10 ± 1.45 D, 0.13 ± 0.82 D, and 2.03 ± 1.91 D, respectively; *P* = 0.002).

### Absolute prediction error according to the IOL formula

The mean absolute refractive prediction error (APE) was 1.10 ± 1.08 diopters (D) using BU II, 1.07 ± 1.00 D using Hoffer-Q, and 1.53 ± 1.32 D using SRKT (*P* = 0.25). A comparison of the mean APE is presented in Table 4.

**Table 4:** Absolute refractive prediction error comparing the different formulas

Factor	Barrett	Hoffer-Q	SRKT	*P*-value
Whole sample APE^2^	1.10 ± 1.08	1.07 ± 1.00	1.53 ± 1.32	0.25
	*N*^1^ = 100	*N* = 31	*N* = 20	
Axial length < 22 mm				
Mean	1.11 ± 1.12	1.10 ± 1.09	1.87 ± 2.00	0.23
*N*	*N* = 59	*N* = 24	*N* = 8	
Axial length ≥ 22 mm				
Mean	1.09 ± 1.03	0.97 ± 0.69	1.31 ± 0.58	0.70
*N*	*N* = 41	*N* = 7	*N* = 12	
Age < 24 months				
Mean	1.73 ± 1.37	1.78 ± 1.59	1.67 ± 1.81	0.99
*N*	*N* = 15	*N* = 7	*N* = 4	
Age ≥ 24 months				
Mean	0.99 ± 0.99	0.87 ± 0.67	1.50 ± 1.25	0.11
*N*	*N* = 85	*N* = 24	*N* = 16	
Average K^3^ < 44.5 diopters				
Mean	1.13 ± 1.19	1.44 ± 1.23	1.18 ± 0.68	0.63
*N*	*N* = 48	*N* = 16	*N* = 12	
Average K ≥ 44.5 diopters				
Mean	1.08 ± 0.97	0.67 ± 0.45	2.06 ± 1.87	0.011*
*N*	*N* = 52	*N* = 15	*N* = 8	
Pairwise comparison	Barrett-SRKT (***P*= 0.04**), Barrett = Hoffer-Q (*P* = 0.56), Hoffer-Q-SRKT (***P*** **= 0.009**)			

Continuous data are presented as the mean ± standard deviation

*N*^1^, number of eyes; *APE*^2^, absolute refractive prediction error; *Average K*^3^, average corneal refractive power

*P-value < 0.05 was considered significant

At mean *k*-values ≥ 44.5 diopters, BU II and Hoffer-Q had an APE lower than SRKT (1.08 ± 0.97 D, 0.67 ± 0.45 D, and 2.06 ± 1.87 D, respectively; *P* = 0.011).

There was no difference in APE upon sub-group analysis by axial eye length or child age.

**Fig. 2:**
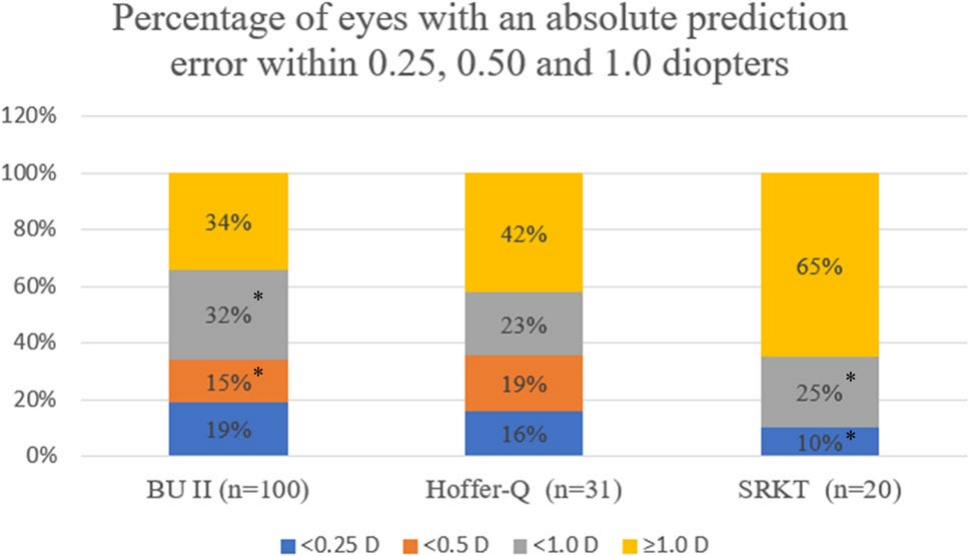
Percentage of eyes with an absolute prediction error within 0.25 diopter (D), 0.50 D, and 1.0 D. n = number of eyes. *P-value < 0.05 was considered significant (significant differences between formulae for various prediction errors are marked with * in the figure)

Percentages of eyes with an absolute prediction error (APE) within 0.25 D, 0.50 D, and 1.0 D from the predicted refractive error are presented in Fig. 2. The SRKT formula was less accurate within 0.5 D (10%) and 1.0 D (35%) compared with BU II (34% and 66%, respectively; *P* = 0.03 and 0.01, respectively).

In eyes with AL ≥ 22 mm, a larger percent of eyes with APE > 0.5 was seen using BU II and Hoffer-Q compared with SRKT (20%, 14%, and 0%, respectively; *P* = 0.02 for the difference between BU II and SRKT). No statistically significant difference was seen for shorter eyes with AL < 22 mm.

In children aged 24 months or older at surgery, a larger percent of eyes with APE > 0.5 was seen using BU II and Hoffer-Q compared with SRKT (16%, 21%, and 0% respectively; *P* = 0.02 for the difference between BU II and SRKT). Also, a larger percentage of eyes with APE > 1.0 was seen using BU II and Hoffer-Q compared with SRKT (29%, 29%, and 25%, respectively; *P* = 0.01 for the difference between BU II and SRKT). No statistically significant difference was seen for children younger than 24 months at surgery.

In eyes with K ≥ 44.5 D, a larger percent of eyes with APE > 1.0 was seen using BU II compared with Hoffer-Q and SRKT (44%, 27%, and 13%, respectively; *P* = 0.04 for the difference between BU II and SRKT). No statistically significant difference was seen for eyes with K < 44.5 D (sub-group analysis of percentages of APE is available as Supplementary Fig. S1).

Univariate linear regression analysis demonstrated a significant correlation between age (*r* =  − 0.28; *p* < 0.001), target refraction (*r* =  − 0.15; *p* = 0.03), and APE. Axial length (*r* =  − 0.02; *p* = 0.41), average K (*r* =  − 0.11; *p* = 0.08), and IOL power (*r* =  − 0.08; *p* = 0.17) were not associated with APE.

Univariate logistic regression analysis showed the odds ratio (OR) for an APE > 1 diopter was 3.6 (*p* = 0.01) with the SRKT formula and 1.4 (*p* = 0.42) with the Hoffer-Q formula relative to BU II. Age was also associated with an APE > 1 D (OR = 0.99, *p* = 0.03), while gender (OR = 0.85; *p* = 0.63), target refraction (OR =  − 1.08; *p* = 0.33), axial length (OR = 1.14; *p* = 0.22), average K (OR = 0.85; *p* = 0.08), in the bag IOL location (OR = 2.00; *p* = 0.10), IOL power (OR = 0.93; *p* = 0.07), and primary IOL insertion (OR = 2.32; *p* = 0.09) were not associated with APE > 1 D. After adjusting to age, multinomial logistic regression analysis showed the odds ratio (OR) for an APE > 1 diopter were 3.45 (*p* = 0.017) with SRKT formula and 1.31 (*p* = 0.52) with Hoffer-Q formula relative to BU II (Table 5).

**Table 5:** Association of Hoffer-Q and SRKT formulas with an absolute prediction error greater than one diopter, relative to Barret formula (*N*^1^ = 151)

Factor	Odds ratio (relative to Barrett)	95% Confidence interval	*P*-value
Formula			
SRKT	3.45	1.24–9.60	0.017*
Hoffer-Q	1.31	0.57–3.04	0.52
Age (months)	0.99	0.98–1.00	0.04*

Odds ratios were analyzed by multinomial logistic regression after adjusting to age

*N*^1^, number of eye

*P-value < 0.05 was considered significant

### Unilateral sub-group analysis (available as Supplementary information S2–S6)

104 eyes of 104 children were included. The mean age at IOL implantation was 5.85 ± 3.9 years (range: 10 months–16.3 years). One month after surgery, the PE was − 0.25 ± 1.61 diopters (D) with BU II, 0.20 ± 1.71 D with Hoffer-Q, and 0.60 ± 1.60 D with SRKT (*P* = 0.15). In eyes with short axial length (AL) < 22 mm, there was no difference in the PE obtained by the three formulae (*P* = 0. 24). In long eyes with AL ≥ 22 mm, BU II had a PE lower than Hoffer-Q (− 0.66 ± 1.41 D and 0.85 ± 1.13 D, respectively; *P* = 0.054).

In children 24 months or older at surgery, BU II had a smaller PE than Hoffer-Q (− 0.40 ± 1.46 D and 0.67 ± 1.11 D, respectively; *P* = 0.007). However, no difference was found in children younger than 24 months at surgery using the three formulae (*P* = 0.30).

In eyes with mean *k*-values ≥ 44.5 D, BU II had a smaller PE than SRKT (− 0.02 ± 1.48 D and 1.68 ± 1.59 D, respectively; *P* = 0.05). An absolute prediction error (APE) of < 1.0 D was obtained in 66% of eyes when using BU II and in 43% of eyes when using SRKT (not statistically significant; *P* = 0.10). The age-adjusted odds ratio (OR) for an APE > 1 D was 2.61 (*P* = 0.09) with the SRKT formula and 2.34 (*P* = 0.11) with the Hoffer-Q formula relative to BU II (defined as OR = 1).

### Sub-group analysis of different biometry and surgical techniques (available as Supplementary information S7)

Sub-group analysis was done for APE of the different formulae stratified by surgical method (performance or anterior vitrectomy and posterior capsulotomy as part of lensectomy with primary IOL implantation) and method of axial length acquisition (IOL master 500 or contact A-scan). No significant differences in the APE were found between the different formulae in this analysis. In eyes that underwent contact A-scan biometry, the mean APE was 1.13 ± 1.13 diopters (D) using BU II, 1.28 ± 1.08 D using Hoffer-Q, and 1.73 ± 1.39 D using SRKT (the number of eyes was 73, 21, and 16, respectively; *P* = 0.17). In eyes measured using IOL master 500, the mean APE was 1.04 ± 0.93 D using BU II, 0.65 ± 0.65 D using Hoffer-Q, and 0.74 ± 0.56 D using SRKT (the number of eyes was 27, 10, and 4, respectively; *P* = 0.43).

In eyes that underwent anterior vitrectomy and posterior capsulotomy as part of lensectomy with primary IOL implantation, the mean APE was 1.07 ± 1.12 D using BU II, 1.31 ± 1.15 D using Hoffer-Q, and 1.13 ± 0.49 D using SRKT (the number of eyes was 64, 17, and 11, respectively; *P* = 0.71). When anterior vitrectomy and posterior capsulotomy were not performed, the mean APE was 1.04 ± 0.93 D using BU II, 0.65 ± 0.65 D using Hoffer-Q, and 0.65 ± 0.66 D using SRKT (the number of eyes was 27, 10, and 3, respectively; *P* = 0.41).

## Discussion

Numerous studies examined IOL power calculation methodology in pediatric eyes (the ones examining BU II are available in Table 1). Nonetheless, there is still a controversy regarding the optimal formula.

Here, we compared the accuracy of IOL power calculation of the BU II formula versus Hoffer-Q and SRKT formulae following lensectomy and IOL implantation in a large pediatric cohort while stratifying the analysis across different ages, axial lengths, and K-readings. Our data showed good accuracy when using the BU II formula. No difference in accuracy was detected overall between the three formulas. Upon sub-group analysis, BU II demonstrated superiority in maintaining good accuracy across changing axial lengths, keratometry values, and different ages. BU II was superior to SRKT’s predictability within 1.0 D or < 0.5 D from target refraction.

In our study, BU II demonstrated a trend toward lower PE compared to Hoffer-Q, especially when compared to SRKT, which was not statistically significant. This finding agrees with several studies that compared BU II with SRKT, Hoffer-Q, and other newer-generation formulae and found comparable PE [7, 11, 12, 14, 15, 17].

Notably, a recent work by Taroni et al. [18] compared the new Hoffer QST formula with the original Hoffer-Q formula and four other latest-generation formulae on 1259 eyes. The new Hoffer QST formula was superior to the original Hoffer-Q formula, especially in very short or long eyes, and achieved results comparable with BU II, EVO, Kane, and RBF (radial basis function) formulae.

When looking at our data, BU II demonstrated a higher percentage of eyes with APE < 1.0 D or < 0.5 D when compared with SRKT. Additionally, the odds ratio (OR) for an APE > 1 diopter was 3.45 with the SRKT formula, compared with BU II. This is in concordance with the study by Elbaz et al. [14] including 68 pediatric eyes with a higher percentage of eyes with APE < 0.5 D using BU II (51.5%), compared with SRKT (31.8%) formula.

Our study shows that shorter eyes with AL < 22 mm, BU II, and Hoffer-Q had a PE lower than SRKT, but the percentage of eyes with APE < 1.0 D or < 0.5 D showed no differences between the formulas. SRKT has been reported to have a better prediction in the eyes of average and high AL in a large adult cohort studied by Aristodemou et al. [19]. However, studies on pediatric eyes demonstrated conflicting evidence regarding the accuracy of SRKT over BU II in small eyes. In a study of 70 pediatric eyes by Yilmaz et al. [9], the BU II formula had the best results in eyes with average AL, while SRK/T and Holladay 1 formulas were better in eyes with shorter AL. Chang et al. studied 68 pediatric eyes and showed that in AL ≤ 21 mm, SRK/T formulas were relatively accurate, while Barrett and Haigis formulas were better in AL > 21 mm [8]. Neither Reitblat et al. [15] nor Elbaz et al. [14] found different accuracy when comparing SRKT and BU II in eyes AL < 22 mm in pediatric eyes. The differences between these studies may stem from numerous factors, including the number of eyes studied, the inclusion of sulcus-implanted IOLs in our research, and the use of contact versus immersion A-scan biometric techniques.

Our study demonstrates that in longer eyes with AL ≥ 22 mm, using the BU II gives a smaller PE compared to Hoffer-Q and a higher percentage of eyes with APE < 0.5 D when compared to SRKT. When using BU II in eyes larger than 22 mm, the PE was smaller than with the Hoffer-Q formula. It has previously been argued that the Hoffer-Q formula tends to be more accurate for small eyes. In an extensive study of 10,277 adult eyes implanted with Sofport IOLs by Aristodemou et al. [19], in eyes with AL between 20 and 21 mm, Hoffer-Q had a larger percentage of eyes with APE < 0.5 (71%), compared with Holladay 1 (52%) and SRKT (36%). In eyes with AL between 27 and 28 mm, Hoffer-Q had a smaller percentage of eyes with APE < 0.5 (56%) compared with SRKT (75%). Chang et al., analyzing 68 pediatric eyes, showed that in eyes with AL > 21 mm, Barrett and Haigis formulas had a larger percentage of eyes with APE < 0.5 (45% and 53%), compared with SRKT (38%), Hoffer-Q (30%), and Holladay I (28%) [8].

Our results show that in eyes with mean K ≥ 44.5, both BU II and Hoffer-Q formulas give a smaller PE when compared to using SRKT and, as well as a larger percentage of eyes with APE < 1.0 D with BU II (44%), compared with SRKT (13%). No difference in mean PE or percentage of eyes with APE < 1.0 D or < 0.5 D was demonstrated between the formulae in eyes with mean K < 44.5.

In agreement with our findings, Eppley et al. [7] found better stability and significantly lower PE for steeper corneas with the BU II formula when compared with Hoffer-Q and Holladay II formulae.

Furthermore, our study shows that in children operated after 24 months of age, using the BU II gave a lower mean PE when compared with Hoffer-Q and SRKT. Also, for children above 24 months of age, the percentage of eyes with APE < 1.0 D or < 0.5 D was higher with BU II when compared with SRKT. We also showed that in children operated before the age of 24 months, no difference in mean PE or the percentage of eyes with APE < 1.0 D or < 0.5 D was detected between the three formulae tested.

In agreement with our findings, Chang et al. [8] found BU II and Haigis formulas to have larger percentages of eyes with APE < 0.5 D (57% and 51%) compared with Hoffer Q (28%) and SRKT (42%), Holladay I (30%) and Holladay II (30%) in children older than two years, and this difference was statistically significant. In contrast to our data, in children younger than two years old, SRK/T formulas were relatively accurate, with 34% of eyes with APE < 0.5 D compared with BU II (18%) and Holladay II (17%); however, this difference was not statistically significant.

Our study explored the accuracy of using the BU II formula versus older generation IOL formulae when calculating IOL power in a large cohort of children. BU II showed good accuracy and superior reliability across variable ages, axial eye lengths, and keratometry values. This information is helpful in daily decision-making as surgeons encounter children with cataracts at varying ages and biometric properties, and the need for one formula to address these different situations is emphasized in the pediatric population.

Nonetheless, the clinical impact of initial post-operative APE on final adult APE is unclear. Pediatric pseudophakic eyes show a myopic shift, which is characterized by significant variability. When an IOL is implanted at younger ages, a more remarkable myopic shift is usually anticipated, and the predictability of final refraction is lower [20]. Oke et al. studied the relative contribution of early post-operative intra-ocular lens (IOL) calculation accuracy on the long-term refractive error in 42 pediatric pseudophakic eyes. Their results showed that early post-operative APE could explain 12% of the variability in the prediction error ten years following surgery [21].

Several limitations should be mentioned. First, bilateral cases were included, which may cause errors in the results due to the similarity in measurements between the two eyes of the same patients and the compounding of the data. However, a sub-group analysis of unilateral cases was done. It showed good performance of BU II with a small PE of − 0.25 ± 1.61 and good reliability across varying axial lengths, K-readings, and ages, as opposed to the older generation formulae (available as Supplementary information S2–S6). Second, the A constant of IOL used in this study was derived from adults due to the lack of an optimized A constant for children. However, the PE of the BU II was close to zero and thus did not need optimization. For the other groups, larger samples are required for constant optimization to be reliable [22]. Third, most axial length measurements were done by contact rather than immersion sonography, which affects accuracy [23]. However, we believe contact A-scan reflects real-world practice in many centers.

Fourth, in contrast to previous studies [7], comparisons of IOL calculations by all formulae for each eye were not made due to a lack of complete anterior chamber depth data.

Fifth, although most children were younger than eight years and thus underwent anterior vitrectomy and posterior capsulotomy as part of lensectomy and IOL implantation, as well as axial length measurement by contact A-scan, some did not. This limitation was addressed by sub-group analysis (see Supplementary Table S7), which showed no significant differences in APE between these sub-groups. It should be mentioned that the sample size of SRKT eyes over eight years old was small.

Sixth, due to the retrospective nature of this work, the groups were not balanced concerning axial length, with more extreme values in the Hoffer and SRKT groups. Also, different IOL types were used in the different groups due to a difference of several years between the BU II group and the other groups (see Table 2).

When calculating the IOL in children, we found that using the Barrett II formula gives an overall good performance with a small mean predictive error. Comparing SRKT, Hoffer-Q, and Barrett formulas shows no significant difference in the PE at one month postoperatively. However, BU II was the only formula to demonstrate stable prediction errors at varying biometric parameters, including axial length, K-readings, and patient ages. As the biometric parameters of the developing eye change with growth, the BU II formula offers a relatively reliable and stable option for pediatric IOL calculation.

We know the significant changes occurring in the developing pseudophakic pediatric eye. We suggest future studies focus on long-term follow-up of the final refraction to improve the accuracy of IOL power calculations in children with cataracts.

### Supplementary Information

Below is the link to the electronic supplementary material.

Supplementary file1 (DOCX 392 KB)

## Data Availability

The corresponding author has full access to all the data in the study and takes responsibility for the data’s integrity, the data analysis’s accuracy, and the decision to submit for publication. Data reported in this work are available upon request from the corresponding author.
