# Supplementary material for: Refractive predictive errors using Barrett II, Hoffer-Q, and SRKT formulae for pediatric IOL implantation
Source: Graefes Arch Clin Exp Ophthalmol. 2024 Feb 15;262(7):2309–20. doi: 10.1007/s00417-024-06401-4 (PMC11222246; doi:10.1007/s00417-024-06401-4)
Supplement: Supplementary file 1 — Supplementary file1 (DOCX 392 KB) [file 417_2024_6401_MOESM1_ESM.docx]

**Refractive predictive errors using Barrett II, Hoffer-Q, and SRKT formulae for pediatric IOL implantation (Supplementary material)**

Or Shmueli, MD^1^, Nur Azem, MD^1^, Ana Navarrete, MD^1^, Milka Matanis-Suidan^1^, MD, Ran David, MD^1^ PhD, Hadas Mechoulam, MD^1^, Irene Anteby, MD^1^

**Correspondence**:

Irene Anteby, MD

ORCID- 0000-0001-6878-0443

Department of Ophthalmology, Hadassah Medical Center

The Hebrew University of Jerusalem, Israel.

Ein-Karem 9112001

E-mail address: irenea@mail.huji.ac.il

**Figure S1. sub-group analysis of percentages of absolute prediction error (APE)**


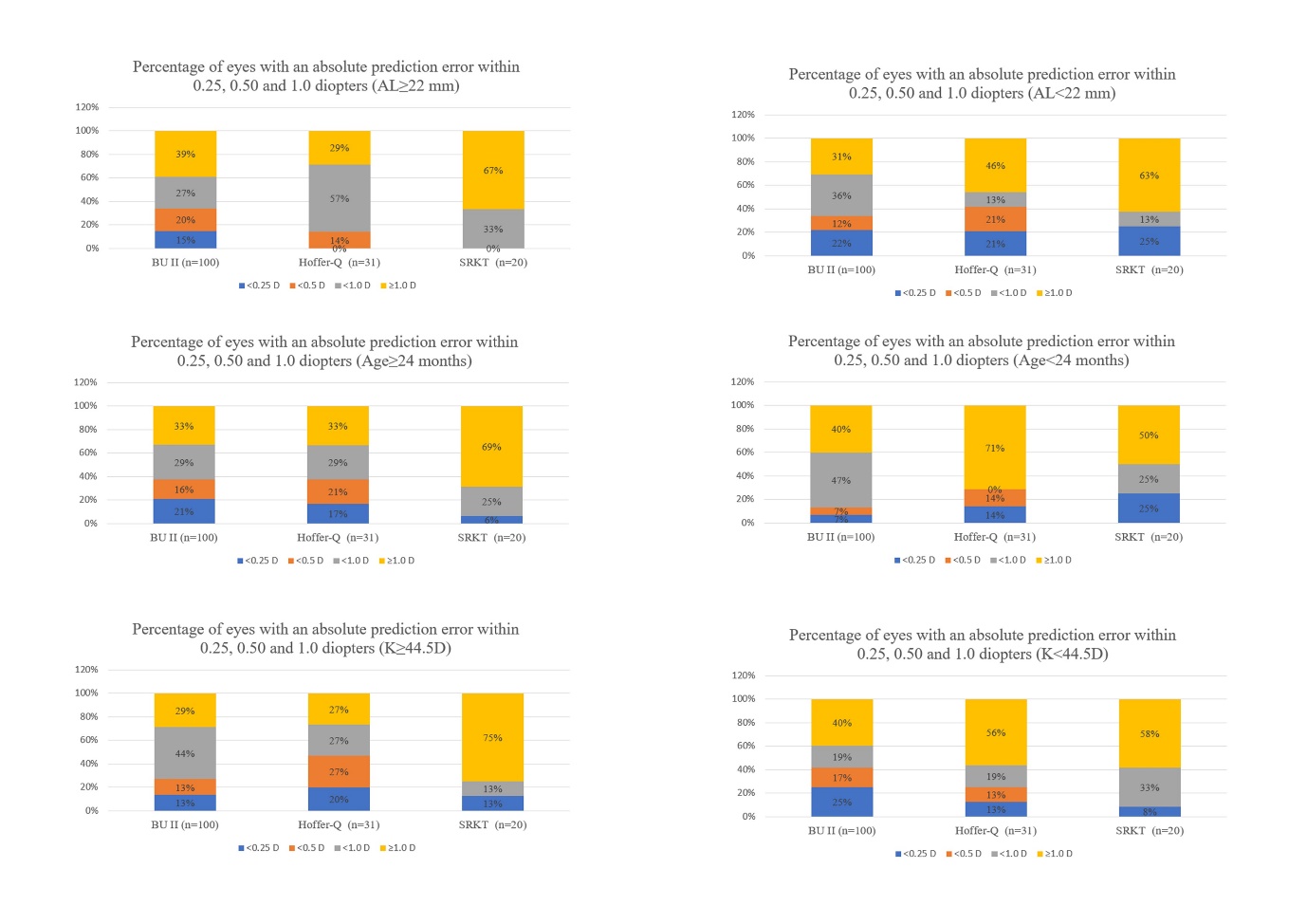


K=Average corneal refractive power; AL=Axial eye length

**Table S2. Baseline patient characteristics (one eye per patient)**

| ***P*-value** | **SRKT (N=14)** | **Hoffer-Q (N=22)** | **Barrett (N^1^=68)** | **Factor** |
| --- | --- | --- | --- | --- |
| 0.76  0.84 | 74.2±47.1 (14-162)  66.5 (27.3-91.0) | 63.7±42.4 (10-130)  61.0 (27.0-101.0) | 71.4±48.6 (10-195)  70.0 (32.25-102.75) | **Age at IOL^2^ implantation (months)**  Mean  Median |
| 0.048 | 85.7% | 45.4% | 55.8% | **Gender (%male)** |
| 1.0 | 0% | 0% | 0% | **Bilateral Cataract**  **(% of eyes)** |
| 0.43 | 86%  14%  0% | 100%  0%  0% | 91%  7%  2% | **Congenital/developmental cataract (%)**  **Traumatic Cataract (%)**  **Associated with PFV^3^ (%)** |
| **0.001**^*^  **0.02**^*^ | 22.8±2.3 (18.2-28.0)  22.6 (21.4-23.5) | 20.9±1.2 (18.4-22.7)  20.8 (19.7-22.0) | 21.8±1.3 (19.5-25.9)  21.6 (20.8-22.7) | **Axial length (mm)**  Mean  Median |
| Difference of means:  Barrett-SRKT (P=0.08, Barrett-Hoffer-Q **(P=0.02)**, Hoffer-Q-SRKT **(P=0.001)**  Difference of medians:  Barrett-SRKT **(P=0.07),** Barrett=Hoffer-Q (P=0.14), Hoffer-Q-SRKT **(P=0.006)** | | | | Pairwise comparison |
| 0.71  0.84 | 44.1±1.9 (40.3-47.4)  43.95 (40.3-46.6) | 44.4±1.9 (39.0-47.8)  44.3 (43.6-45.4) | 44.5±1.8 (39.7-48.3)  44.5 (43.4-45.7) | **Average K^5^ (D^4^)**  Mean  Median |
| 0.17 | 28.6% | 13.6% | 10% | **Secondary IOL**  **insertions (%)** |
| 0.25 | 64.2% | 86.3% | 82% | **IOL location**  **(% located in the bag)** |
| **<0.0001** | Acrysof IQ (57%)  Alcon 3 piece (43%) | Acrysof IQ (86.3%)  Alcon 3 piece (13.6%) | B&L MX60 (81%)  AR40E 3 piece (10%)  Matrix 3 piece (6%)  Acrysof IQ (1%)  Alcon 3 piece (0%)  PMMA (1%) | **IOL type (%)** |

Continuous data are presented as the mean ± standard deviation (Minimum-maximum) and median (inter-quartile range).

Categorical data are presented as proportions.

N^1^=number of eyes; IOL^2^=Intra-ocular lens implant; PFV^3^=persistent fetal vasculature; D^4^=Diopters; Average K^5^=Average corneal refractive power

^*^ P-value<0.05 was considered significant.

**Figure S3. Comparison of Prediction error (PE) between the different IOL formulae (one eye per patient)**


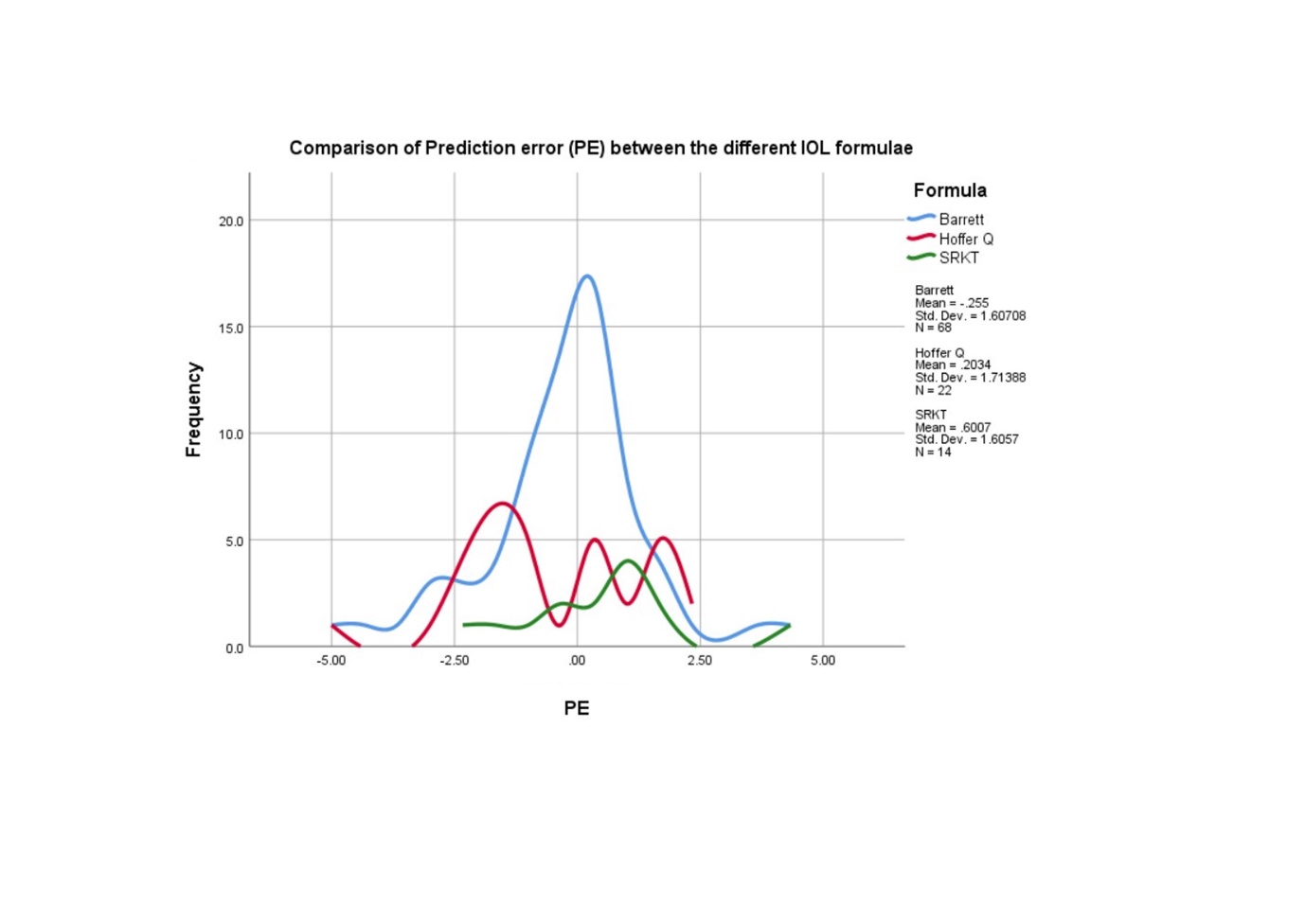


The mean refractive prediction error was -0.25±1.60 with BU II, 0.20±1.71 with Hoffer-Q, and 0.60±1.60 with SRKT (P=0.15).

Data are presented as the mean ± standard deviation

N=number of eyes; PE=refractive prediction error; IOL=intra-ocular lens implant

**Table S4. Comparison of refractive prediction error using the different formulas (one eye per patient)**

| ***P*-value** | **SRKT** | **Hoffer-Q** | **Barrett** | **Factor** |
| --- | --- | --- | --- | --- |
| 0.15 | 0.60±1.60  N=14 | 0.20±1.71  N=22 | -0.25±1.61  N**^1^**=68 | **Whole sample PE^2^** |
| 0. 24 | 1.40±1.76  N=5 | 0.01±1.83  N=17 | 0.03±1.69  N=40 | **Axial length<22 mm**  Mean  N |
| **0.054*** | 0.16±1.41  N=9 | 0.85±1.13  N=5 | -0.66±1.41  N=28 | **Axial length≥22 mm**  Mean  N  Pairwise comparison |
| Barrett-SRKT (P=0.28), Barrett-Hoffer-Q **(P=0.07)**, Hoffer-Q-SRKT (P=0.64) | | | |  |
| 0.30 | 0.84±3.03  N=3 | -1.39±2.52  N=5 | 0.38±2.07  N=13 | **Age<24 months**  Mean  N |
| **0.007*** | 0.53±1.22  N=11 | 0.67±1.11  N=17 | -0.40±1.46  N=55 | **Age≥24 months**  Mean  N  Pairwise comparison |
| Barrett-SRKT (P=0.10), Barrett=Hoffer-Q **(P=0.01)**, Hoffer-Q-SRKT (P=0.96) | | | |  |
| 0.45 | 0±1.33  N=9 | 0.22±2.19  N=12 | -0.48±1.71  N=34 | **Average K<44.5 diopter**  Mean  N |
| **0.050*** | 1.68±1.59  N=5 | 0.18±0.99  N=10 | -0.02±1.48  N=34 | **Average K^3^≥44.5 diopter**  Mean  N  Pairwise comparison |
| Barrett-SRKT (**P=0.04**), Barrett=Hoffer-Q **(**P=0.91**)**, Hoffer-Q-SRKT (P=0.14) | | | |  |

Continuous data are presented as the mean ± standard deviation.

N^1^=number of eyes; PE^2^=refractive prediction error; Average K^3^=Average corneal refractive power

^*^ P-value<0.05 was considered significant.

**Table S5. Association of Hoffer-Q and SRKT formulas with a refractive prediction error greater than 1 diopter, relative to Barret formula (N**^1^**=104) (one eye per patient)**

| **P-value** | **95% Confidence interval** | **Odds ratio**  **(Relative to Barrett)** | **Factor** |
| --- | --- | --- | --- |
| 0.11  0.09 | 0.81-8.42  0.88-6.24 | 2.61  2.34 | **Formula**  SRKT  Hoffer-Q |
| **0.04** | 0.98-1.00 | 0.99 | **Age (months)** |

Odds ratios were analyzed by binary logistic regression.

N^1^=number of eyes;

^*^ P-value<0.05 was considered significant.


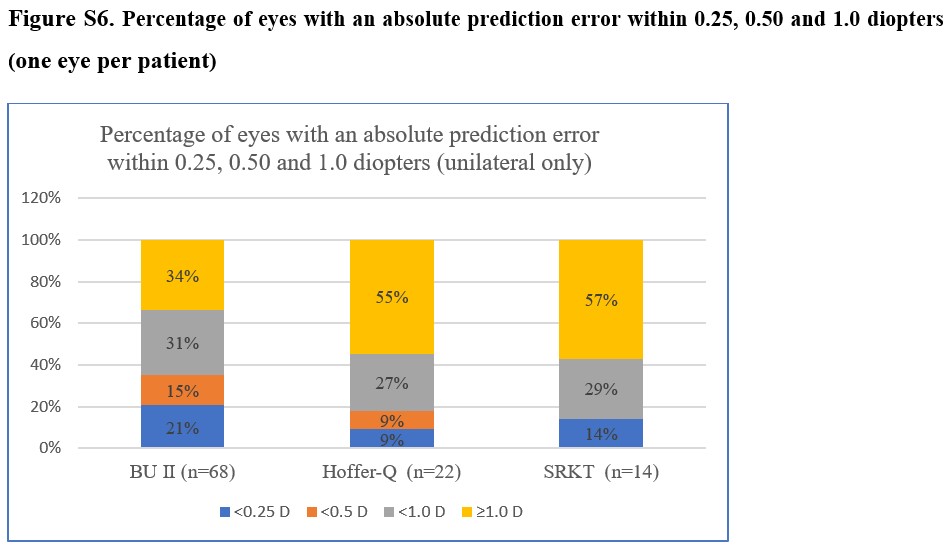


D=Diopters; n=number of eyes

**Table S7. Comparison of absolute prediction error using the different formulas- additional sub-group analysis of different surgical and biometric methods**

| ***P*-value** | **SRKT** | **Hoffer-Q** | **Barrett** | **Factor** |
| --- | --- | --- | --- | --- |
| 0. 17 | 1.73±1.39  N=16 | 1.28±1.08  N=21 | 1.13±1.13  N=73 | **Axial length by A-scan**  Mean APE^1^ (D^2^)  N^3^ |
| 0.43 | 0.74±0.56  N=4 | 0.65±0.65  N=10 | 1.04±0.93  N=27 | **Axial length by IOL master 500**  Mean APE  N |
| 0. 71 | 1.13±0.49  N=11 | 1.31±1.15  N=17 | 1.07±1.12  N=64 | **Surgery with anterior vitrectomy and posterior capsulotomy**  Mean APE  N |
| 0.41 | 0.65±0.66  N=3 | 0.65±0.65  N=10 | 1.04±0.93  N=27 | **Surgery without anterior vitrectomy and posterior capsulotomy**  Mean APE  N |

Sub-group analysis for APE of the different formulae stratified by surgical method (performance or anterior vitrectomy and posterior capsulotomy as part of lensectomy with primary IOL implantation) and method of axial length acquisition (IOL master 500 or contact A-scan).

Continuous data are presented as the mean ± standard deviation.

APE^1^=Absulote prediction error; D^2^=Diopters; N^3^=number of eyes.

^*^ P-value<0.05 was considered significant.
